# Nanotechnology as a Shield against COVID-19: Current Advancement and Limitations

**DOI:** 10.3390/v13071224

**Published:** 2021-06-24

**Authors:** Mahendra Rai, Shital Bonde, Alka Yadav, Arpita Bhowmik, Sanjay Rathod, Pramod Ingle, Aniket Gade

**Affiliations:** 1 Nanobiotechnology Lab., Department of Biotechnology, Sant Gadge Baba Amravati University, Amravati 444 602, Maharashtra, India; shitalbonde@gmail.com (S.B.); nanoalka@gmail.com (A.Y.); pingle23@gmail.com (P.I.); aniketgade@sgbau.ac.in (A.G.); 2 Faculty of Medicine, Dentistry and Health, The University of Sheffield, Sheffield S10 2TN, UK; arpitabhowmik035@gmail.com; 3 Department of Immunology, University of Pittsburgh, Pittsburgh, PA 15261, USA; SBR21@pitt.edu

**Keywords:** COVID-19, SARS-CoV-2, nanotechnology, detection, treatment

## Abstract

The coronavirus disease 2019 (COVID-19) caused by severe acute respiratory syndrome coronavirus 2 (SARS-CoV-2) is a global health problem that the WHO declared a pandemic. COVID-19 has resulted in a worldwide lockdown and threatened to topple the global economy. The mortality of COVID-19 is comparatively low compared with previous SARS outbreaks, but the rate of spread of the disease and its morbidity is alarming. This virus can be transmitted human-to-human through droplets and close contact, and people of all ages are susceptible to this virus. With the advancements in nanotechnology, their remarkable properties, including their ability to amplify signal, can be used for the development of nanobiosensors and nanoimaging techniques that can be used for early-stage detection along with other diagnostic tools. Nano-based protection equipment and disinfecting agents can provide much-needed protection against SARS-CoV-2. Moreover, nanoparticles can serve as a carrier for antigens or as an adjuvant, thereby making way for the development of a new generation of vaccines. The present review elaborates the role of nanotechnology-based tactics used for the detection, diagnosis, protection, and treatment of COVID-19 caused by the SARS-CoV-2 virus.

## 1. Introduction

Coronavirus disease (COVID-19) or SARS-CoV-2 infection is caused by a virus that belongs to the subfamily Coronavirinae (family: Coronaviridae). The disease emerged at the end of 2019 in the city of Wuhan, China. The virus is spherical, enveloped with spike-like proteins protruding from the virion surface, and has a single-stranded RNA genome. The virus has approximately 79% genomic similarity with the severe acute respiratory syndrome coronavirus (SARS-CoV) and 50% genomic similarity with the Middle East respiratory syndrome coronavirus (MERS-CoV) [1]. SARS-CoV-2 has spread fast worldwide, causing a global pandemic outnumbering the people infected by either SARS-CoV or MERS-CoV since their emergence in 2002 and 2012, respectively. The clinical manifestation of the virus includes fever, dry cough, loss of taste and smell, body pain, anorexia, dyspnea, fatigue, and life-threatening acute respiratory distress syndrome (ARDS) [1]. Although lungs are the primary target of the virus, other systems such as cardiovascular, kidney, liver, central nervous system, and the immune system are also compromised in COVID-19 [2]. As the virus continues to spread in an implacable way causing widespread social, health, and economic disruptions, preventive measures such as social distancing, washing hands, and wearing masks have become pertinent to contain viral transmission. With no official drugs approved for the disease, the current treatment mainly involves symptomatic relief coupled with respiratory support for more severe patients. The heterogeneous nature of the disease and constant mutation in the virus warrants a need for diagnostic tools.

In this regard, nanotechnology is being seriously investigated for its potential in the development of therapeutics, vaccines, diagnostic techniques, and strategies to reduce the healthcare burden. The unique properties of nanoparticles such as their small size, enhanced solubility, better target reachability, improved half-life, reduced side-effects, and surface adaptability are being utilized to bring out a much-needed clinical transformation that could be effective directly against the virus [3,4]. Researchers are now looking into nanotechnology for developing improved assays and nanosensor-based diagnostic techniques, improved delivery of medications, and increased circulation time of the drugs. Thus, nanotechnology seems to hold the potential to bring in innovative alternatives effective against the virus.

## 2. Risk of Comorbidities in SARS-CoV-2 Infection

Association between the clinical phenotype of COVID-19 and pre-existing chronic co-morbidities is currently poorly described in the literature and is broadly based on small retrospective studies. As per CDC statistics, 80% of COVID-19-related deaths and 47% of hospitalizations occurred in people above 65 years, and co-morbidities played a major contributing factor. The increasing number of patients, poor prognosis in a certain population, and a limited number of medical supplies have overwhelmed the existing healthcare system. Greater understanding of the correlation of risk factors and the disease progression of COVID-19 could help in disease management by personalizing the treatment for improved outcomes. Furthermore, classifying patients into severe and non-severe groups could reduce the healthcare burden.

Co-morbidities including cardiovascular, cerebrovascular, neurological diseases, cancer, and diabetes are closely associated with COVID-19-related ICU admissions and deaths [5,6]. As per a meta-analysis conducted by Khamseh et al., pre-existing cardiovascular diseases increased the risk of severe COVID-19 significantly by 4.8-fold [5]. COVID-19, along with viral pneumonia, has been reported to cause cardiovascular manifestations such as myocardial injury, arrhythmias, myocarditis, and thromboembolism coexisting with the increased amount of cardiac troponin I and c-reactive protein in patients with pre-existing cardiovascular disease [7]. On infection, the virus causes hyperinflammation by over-producing pro-inflammatory cytokines. This hyperinflammation/cytokine storm was seen to further lead to abnormalities in the coagulation system, affecting multiple organs [7]. The potential drug–disease interaction could also contribute to cardiovascular complications from the disease. For example, currently prescribed drugs for COVID-19 such as hydroxychloroquine and azithromycin have pro-arrhythmic effects.

Diabetes, more prevalent in the older population, also increases the risk of developing severe COVID-19. The possible factors may include high glucose levels, impaired metabolic control, low-grade chronic inflammatory state, and impaired coagulation [8]. According to an estimate, 30-day mortality in cancer patients with COVID-19 was 13–33% compared with 0.5–2% in the general population [9]. These statistics were subject to stage, and type of cancer, such as hematological malignancies, were observed to have a worse prognosis over solid tumors. Additionally, in cancer, treatment-related outcomes such as immunosuppression and anemia weakens the body’s ability to fight off the disease.

The severity of COVID-19 was closely associated with cerebrovascular and neurological diseases [5]. Along with the respiratory tract, the virus is seen to invade the nervous system, indicated by the loss of smell, taste, and impaired consciousness in the early stage of the infection. An increasing number of studies report delirium, seizures, and encephalopathy as an outcome of the disease, further hinting at its effects in the brain. An independent study conducted by the scientists in the City of Hope, USA, discovered that the ApoE4 gene, known to increase the risk of developing Alzheimer’s, was also associated with increased susceptibility and severity of COVID-19 [10]. The ApoE4 gene plays a vital role in modulating the pro-inflammatory activity of the macrophages. Thus, it can be predicted that Alzheimer’s patients are at an increased risk of hyperinflammation leading to cytokine storm and severe COVID-19. The damaged blood–brain barrier (BBB) in patients with Alzheimer’s and dementia, particularly vascular dementia, predisposes them to the infection, as the brain is more easily accessible to the virus in such patients [11]. Memory impairment makes it even more difficult for these patients to comply with preventive measures, such as wearing masks, sanitizing hands, maintaining social distance, amongst others, increasing their risk of contracting the disease. Early detection is the key in COVID-19, which is not possible in such patients, adding to the risk.

## 3. Mechanism of Immune Response after Infection of SARS-CoV-2

Generally, the immune system is the best defense mechanism against viruses such as SARS-CoV-2 and other pathogens (bacteria, fungi, and protozoans) by clearing the infections or destroying the virus-infected cells. SARS-CoV-2 primarily comes into circulation via respiratory droplets and additionally through aerosol, direct contact with contaminated surfaces, and fecal–oral transmission [12,13,14]. The SARS-CoV-2 arrives at the host cells via the respiratory tract, airway, alveolar epithelial cells, vascular endothelial cells, and alveolar macrophages [15,16,17]. These cells initiate an early virus infection and consequent replication due to their expression of the ACE2 receptor needed for SARS-CoV-2 entry [18]. Contrary to the typical common cold to moderate upper-respiratory illness observed in coronaviruses, the novel SARS-CoV-2 causes severe “flu-like” signs that can proceed to pneumonia, acute respiratory distress (ARDS), renal failure, and in some cases death [19,20,21,22].

Once SARS-CoV-2 enters the target cell, the host immune system identifies the whole virus or antigenic parts such as spike proteins and provokes both arms of the immune system (innate and adaptive). Like many other RNA viruses, the recognition of SARS-CoV-2 begins with the detection of its genome by host pattern recognition receptors (PRRs), which signal downstream via recruited adaptor proteins, ubiquitin ligases, and kinases, culminating in transcription factors and the ultimate expression of immune genes, including IFNs, cytokines, and chemokines. The IFN-signaling pathway is frequently a primary target of evasion due to its rapidity and effectiveness in eliminating viral infection. SARS-CoV-2 is highly sensitive to IFN responses and acts at several levels in these pathways to antagonize mammalian immune recognition, intruding with downstream signaling or inhibiting specific IFN-stimulated gene (ISG) products [23]. Severe SARS-CoV-2-infected patients trigger hyperimmune responses with high intensities of inflammatory cytokines/chemokines but not enough antiviral cytokine interferon beta (IFN-β) or interferon lambda (IFN-λ), leading to persistent viremia [24]. SARS-CoV-2 most potently inhibits type I and type III IFN expression in the bronchial epithelial cells of both humans and ferrets [25].

As we know, the adaptive immune response against any viral infection is the key to disease severity; T cells especially are central players in the immune response to viral infection. An enhanced understanding of human T cell-mediated immunity in COVID-19 is vital for optimizing therapeutic and vaccine strategies. The immune system, i.e., the innate and acquired immune response, is activated by SARS-CoV-2 infection. Several studies evaluating the clinical features of SARS-CoV-2-infected patients have reported an incubation time of 4–7 days before the onset of symptoms and an additional 7–10 days before the development of severe COVID-19 [26]. After SARS-CoV-2 entry into the host, a virus attaches to cells expressing ACE2, which facilitates its replication. The viral peptides present through major histocompatibility complex (MHC) class I proteins expressed by antigen-presenting cells (APC) such as dendritic cells (DC) to the cytotoxic CD8+ T cells [27].

**Figure 1:**
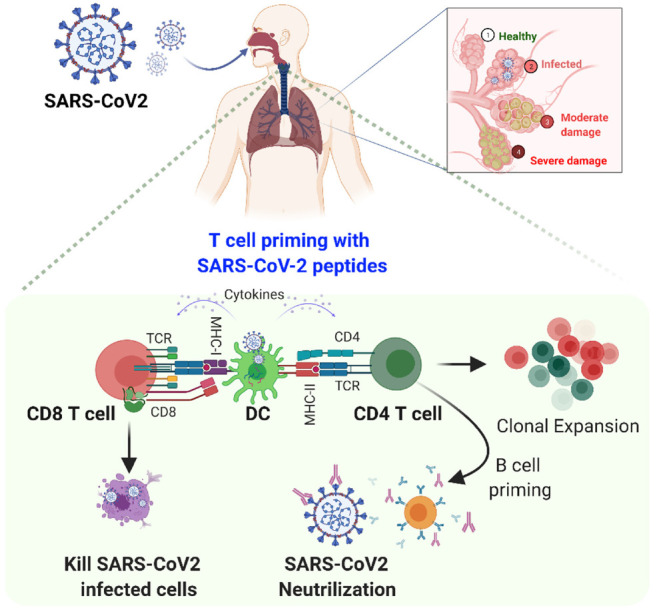
COVID-19 and host immune responses. Following inhalation of SARS-CoV2 into the respiratory tract, the virus traverses deep into the lower lung, where it infects a range of cells expressing its receptor ACE2, including alveolar airway epithelial cells, vascular endothelial cells, and alveolar macrophages. In the innate arm, immune cells primarily recognize the viral RNA by their receptors, such as Toll-like receptors (TLRs) that signal downstream to produce type-I/III interferons (IFNs) and pro-inflammatory mediators as the first line of defense. Furthermore, IFN triggers JAK/STAT signaling to activate interferon stimulating genes (ISGs) to fight SARS-CoV2. In the adaptive arm, the viral peptides are presented through major histocompatibility complex (MHC) class I proteins expressed by dendritic cells (DC) to CD8 T cells; these cells directly kill the virus-infected cells. Further, helper CD4+ T cells are activated through MHC-class II and differentiate B cells into plasma cells (antibody-producing cells) and memory cells. These SARS-CoV2 specific antibodies can neutralize the virus. Overall, both cells play an important role in eradicating SARS-CoV2 from the host.

Further, these cytotoxic CD8+ T cells activate and expand to initiate virus-specific effector and memory phenotypes. For a quick response, the viral antigens are recognized by APCs, such as DC and macrophages, which present viral epitope to helper CD4+ T cells through MHC-Class-II molecules. By stimulating antibody-producing B cells to produce anti-SARS-CoV-2 antibodies such as anti-SARS-CoV-2 IgM, IgA, and IgG, B cells can directly identify the viruses, get activated by them, and interact with helper CD4+ T cells. The first antibody secretion, i.e., IgM isotype primary virus-specific antibody response, is observed within the first week following symptoms. IgG isotype antibodies response comes after the initial IgM response, which mostly retains a lifelong immunity (Figure 1).

Although most COVID-19 patients recover from the mild and moderate disease within a week, some individuals develop severe pneumonia in the second week, shadowed by cytokine storm within the third week of the illness. The cytokine storm is a multifaceted network of extreme molecular events integrated by clinical characteristics such as systemic inflammation and multiorgan failure. Cytokine storm is encouraged by the activation of huge numbers of white blood cells, including B cells, T cells, macrophages, dendritic cells, neutrophils, monocytes, NK cells, and resident tissue cells (epithelial and endothelial cells), which secrete high quantities of pro-inflammatory cytokines [28]. Overall, both innate and adaptive systems play an important role in eradicating the SARS-CoV2 from the host.

## 4. State-of-the-Art of Nanomaterials as Anti-SARS-CoV-2

The recent surge of coronavirus known as SARS-CoV-2 has widely spread across the world; the efficiency of traditional treatment systems also faded due to the emergence of new strains and viral mutations. To overcome the limitations of conventional systems, an improved multidisciplinary approach is needed. Nanomaterials in the form of detection and diagnostic tools, protection equipment, and disinfecting agents can provide much needed protection against SARS-CoV-2.

### 4.1. Nanobiosensors

Although serology-based tests and reverse transcription-polymerase chain reaction (RT-PCR) are routinely used for the detection of COVID-19, there is a need for accuracy and rapidity in diagnosis that can be fulfilled by the use of ultrasensitive nanobiosensors that play a major role in the detection of novel coronavirus. Nanobiosensors provide a rapid, cost-effective, accurate, and miniaturized platform for the detection of SARS-CoV-2 [29].

#### 4.1.1. Affinity-Based Nanobiosensor

Affinity-based nanobiosensors demonstrate the high specificity of bioreceptors, such as antibodies, ssDNA, and aptamers with nanoparticles, which lead to enhanced sensitivity and lower detection limits. Gold nanoparticles, gold nanoislands, graphene, and nanowires are employed for the detection of coronavirus. Gold nanoparticles conjugated with carbon nanotubes improve binding capacity and efficient immobilization matrix. Gold nanoislands are aggregates of gold with a dimension of 20–80 nm and are synthesized by deposition at annealing of gold nanoparticles at elevated temperature for several hours, and these gold nanoislands are also utilized for sensing application [30].

#### 4.1.2. Optical Nanobiosensor

Carbon nanotubes, gold nanoislands, and graphene are majorly used in optical and electrochemical biosensors. Gold nanoislands made of tiny gold nanostructures can be developed with artificially synthesized DNA receptors and complementary RNA sequences of SARS-CoV-2 on a glass substrate. As COVID-19 is a single-stranded RNA virus, the receptor of the nanobiosensor acts as a complementary sequence to the RNA sequence of the coronavirus and detects the virus. LSPR (localized surface plasmon resonance) was used to detect RNA sequence binding to the sensor. After binding of the molecules on the surface of the nanobiosensor, the local infrared index changes and an optical nanobiosensor measures the changes and identifies the presence of RNA strands [28].

Nanobiosensors are used to detect COVID-19. It includes the use of antibodies or cDNA to carefully encapsulate viral RNA. A grapheme-based FET (field effect transistor) device is used for the determination of SARS-CoV-2 viral load in nasopharyngeal swabs of COVID-19 patients. The graphene-based FET nanobiosensor consists of a graphene sheet as the sensing area, transferred to a SiO_2_/Si substrate and SARS-CoV-2 spike antibody immobilized on the graphene sheet. The biosensors help detection of SARS-CoV-2 antigen spike even at the concentration of 1 fg/mL in phosphate buffer [30].

#### 4.1.3. Electrochemical Nanobiosensors

Electrochemical sensors are highly sensitive and could be easily miniaturized. Modified electrochemical biosensors in combination with gold nanoparticles show improved applications and can be used for the detection of MERS-CoV. The nanobiosensor is designed with a group of carbon electrode-coated gold nanoparticles. In one study, it was observed that the recombinant spike (S1) protein gets immobilized to gold nanoparticles and competes with the virus particles for binding to the antibody. When there is an absence of virus infection, it binds to the immobilized spike protein. As this nanobiosensor system possesses a group of electrodes, it can be utilized to detect different coronaviruses [31].

Electrochemical nanobiosensors can also be used for the identification of viral nucleic acids [32]. An electrochemical genosensor developed for the detection of SARS was designed by using a monolayer of thiolated oligonucleotides self-assembled on gold nanoparticles-coated carbon electrodes. The oligonucleotide sequences are specific to nucleocapsid protein of SARS, and the viral infection is detected through enzymatic amplification of viral DNA. The nanobiosensor helps the highly sensitive detection of SARS [32]. An electrochemical nanobiosensor fabricated using gold nanoparticles modified with a carbon electrode and recombinant spike protein S1 as biomarker was developed for the detection of MERS-CoVs; however, this technique also holds promise for the detection of coronaviruses. The biosensor was developed using fluorine-doped substrate and gold nanoparticles as a signal amplifier due to its electrical conductivity [33].

#### 4.1.4. Chiral Nanobiosensors

Chiral nanobiosensors provide rapid detection and hence are very useful in distinguishing SARS-CoV-2. Zirconium quantum dots and magnetic nanoparticles in conjugation with coronavirus-specific antibodies bind to the viral target and form magneto plasmonic-fluorescent nanohybrids that could be separated by an external magnet using the optical detection technique. The nanobiosensor showed application in the detection of various virus cultures, including coronaviruses [31].

Ahmed et al. [34] reported a self-assembled technique for the development of a chiral immunosensor using gold nanoparticles and quantum dots. The immunosensor showed detection of virus infection such as adenovirus, avian influenza virus, and coronavirus using blood samples. For the study, virus samples were added to antibody-conjugated chiral gold nanoparticles associated with antibody-conjugated quantum dots. Circular dichroism was used for measuring chiro-optical response.

#### 4.1.5. Nanoimaging System

The Oxford Nanoimaging system can be used for the detection of fluorescently labeled coronaviruses. This system was developed by the scientists from the Department of Physics, University of Oxford. It is an extremely rapid test for the detection of coronavirus. This innovative technology does not require lysis, purification, or amplification process and yields results in 5 min. The technique involves taking direct throat swabs of infected persons and rapid labeling of the virus particles in the sample with short fluorescent DNA strands; the nanoimaging system and machine learning software rapidly detects the virus [35].

### 4.2. PPE (Personal Protective Equipment) Kits

**Figure 2:**
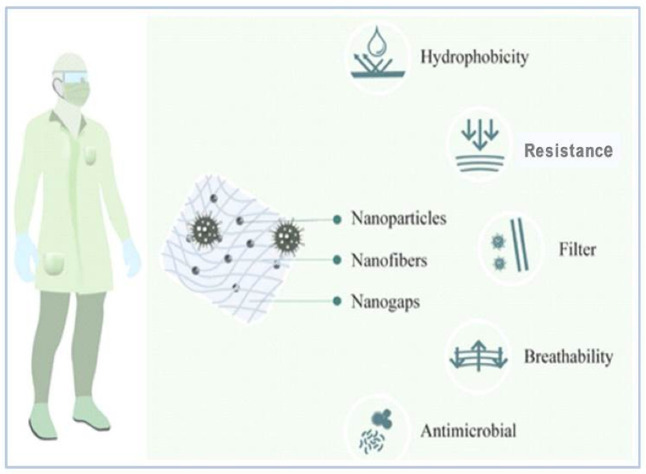
Nanotechnology applications for preparation of PPE Kit [36].

One of the major reasons for widespread COVID-19 infection is person-to-person contact and the respiratory droplets of the infected person. The healthcare professionals need to use appropriate PPE kits, masks, and gloves to protect themselves from the infection. In such difficult circumstances, nanomaterials prove to be an efficient aid for biological and chemical protection. Nano-engineered facemasks, gloves, and PPE kits provide comfortable, hydrophobic, and antimicrobial activity without altering the texture of a fabric (Figure 2) [36]. PPE kits work as an effective barrier against airborne droplets. The use of nanomaterials with textile fibers can provide antimicrobial properties in textile. For example, nano-silver (AgNPs)-impregnated fabrics have already demonstrated antimicrobial properties. AgNPs-based face masks, smocks, lab coats, hospital curtains, etc. have proved to be highly antimicrobial. In this context, the controlled release of nanoparticles for a longer time can serve in modulating the antiviral properties of the fabric [31].

Bhattacharjee et al. [37] reported the use of graphene with silver or copper nanoparticles to enhance the antimicrobial activity of PPE fabric. Graphene on incorporation in a fabric can improve its mechanical strength, antimicrobial property, flame resistance, and the flexibility of the fabric. Metal nanoparticles including silver, copper, and titanium can be associated with graphene to improve its antimicrobial activity, conductivity, and durability. Medical aprons and PPE kits engineered using nanomaterials provide enhanced applications such as hydrophobicity, enhanced antimicrobial activity, and breathability. Hydrophobic nanowhiskers made up of billions of hydrocarbons are extremely small compared to cotton fibers, and they prevent the absorption of droplets. Engineered nanoparticles enhance the surface of textiles and inhibit the growth of pathogenic micro-organisms. Quaternary ammonium salts, polymers, or peptides at the nanoscale level prevent the oxidation of microbial membranes and control their growth [36].

### 4.3. Nanomasks

One of the most important techniques for prevention against viruses is the use of face masks, as it is crucial for both the infected and non-infected individuals to prevent virus transmission. Various textile products are used for the preparation of facemasks coated with nanoparticles with antiviral properties [38].

Campos et al. [36] also highlighted the use of nanocoated masks for better protection. Nanoparticles do not affect the hydrophobicity and breathability of the fabric. For example, silver and copper metal nanoparticles could be incorporated with different fabrics such as cotton, polyester, polyamide, and cellulose-based fabric to strengthen their use as a filter and also as potential antimicrobial agents. Face masks coated with silver and copper nanoparticle dual-layer coatings have also been designed.

Preliminary studies have demonstrated that silver nanoparticles and silica composite nanocoatings can protect from the lethal effect of SARS-CoV-2. Respiratory face masks incorporating nanoparticles are enhanced owing to the virucidal properties of the nanoparticles. Scientists from the Queensland University of Technology, Australia, have designed a facemask from cellulose nanofibers that can filter particles smaller than 100 nm and is breathable with disposable filter cartridge. Additionally, LIGC Applications Ltd. USA has developed a reusable mask using microporous conductive graphene foam, which traps microbes, and the conduction of electrical charge kills the pathogenic micro-organisms [39]. Nanocellulose nanofibers obtained using plant waste material are claimed to be used for the development of orthogonally aligned nanofiber-based face masks. Nanofibers were produced using insulation and a block electro spinning technique. The orthogonal design of the nanofibers minimized the pressure towards the air filter, enhancing the filtration effect. The nanofiber-based facemasks were water-resistant, had high filtration capacity, and were effective after multiple washes [38].

### 4.4. Sanitizers and Disinfectants

Viruses are capable of spreading disease and have the capability of becoming a pandemic; however, technological innovations in the field of nanotechnology significantly help in overcoming viruses. Metal nanoparticles such as silver, copper, and titanium show antiviral activity and can be used as an alternative to chemical disinfectants for protection against SARS-CoV-2. [39]. Environmentally friendly, non-irritating nanosilver-based multiuse sanitizer has been introduced using a nanocolloidal technique. The sanitizer shows effective antiviral, antibacterial, and antifungal activities. NanoTechSurface, Italy, has also developed a disinfectant solution based on silver ions and titanium dioxide for disinfecting surfaces contaminated with coronavirus. The nanopolymer-based disinfectant also shows effective antimicrobial activity, is easy to develop, and is cost-efficient, non-inflammable, and biodegradable. This kind of disinfectant has benefits over chemical-based disinfectants as they are biodegradable and do not catch fire. Wero Water Services has designed biopolymer-based disinfectants that are used by the Prague Public Transit Company for sanitization of public transport vehicles [38].

### 4.5. Antiviral Coatings

Bio-contamination of surfaces and medical devices is a growing concern amid the coronavirus pandemic. The virus-laden respiratory droplets of COVID-19 patients, when exposed in air, deposit on various surfaces and get transmitted to humans; such virus-infected surfaces are known as “fomites” and serve as infectious agents in the transfer of the virus. Traditional disinfecting techniques provide temporary protection, and the bio-burden returns to its original form in a short time span. Non-migratory quaternary ammonium cations (QUATs) and positively charged silver nanoparticles dispensed in polymer matrix can be used for the production of antimicrobial coatings. This coating surface repels oil and water and inactivates coronavirus. It is proposed that silver nanoparticles can inhibit replication of virus nucleotides and inactivate SARS-CoV-2 by interacting with surface spike proteins [40].

Super-hydrophobic nanocoatings could also be used to prevent the transmission of viruses. Copper nanoparticles show antibacterial and antiviral properties and are used to develop super-hydrophobic nanocoatings through the dispersion of nanoparticles in a flexible polymer matrix with the help of a solvent such as acetone. The resultant emulsion can be spray-coated on different surfaces such as doors, knobs, wooden surfaces, and fabrics [40].

Copper and titanium bilayer coatings can be used as nanocoatings over glass surfaces. Silver nanoparticles have also been employed to coat stainless steel surfaces, as most medical devices are made of stainless steel. The synthesis of lysozyme–silver nanoparticles and electrophoretically depositing them on the surface of instruments such as scalpel blades has recently been reported [41]. Erkoc and Uluchan-Karnak [42] demonstrated the use of silver, gold, magnesium oxide, copper oxide, titanium oxide, and zinc oxide nanoparticles to produce coatings with antimicrobial properties. Copper nanoparticles and cardboard materials prevent SARS-CoV-2 infection more efficiently compared with stainless steel and plastic surfaces.

### 4.6. 3D-Printing

**Figure 3:**
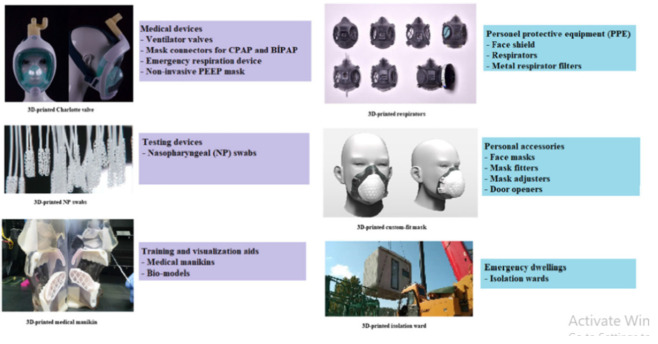
Equipment that could be 3D printed [43].

3D printing, also known as additive manufacturing or rapid prototyping, is basically a production technology that utilizes materials such as plastic or metal stacked in 3D layers to create 3D products. 3D printing is mostly used in the field of engineering. It is also used extensively in the healthcare industry. 3D printed face masks, PPE kits, face shields, auxiliary accessories, door openers, and pushbuttons have been designed and offer great opportunities. However, there are several challenges in 3D printing that have to be answered through future research and technology [43,44]. Coronavirus infection can be divided into three stages: asymptomatic incubation period and severe and non-severe symptomatic period. When the defense system of the patient is unable to fight infection, disruption of the tissues occurs, affecting the kidney and intestine and causing inflammation in lungs. 3D printing technology could be used for the production of simple, inexpensive, and structured drug delivery systems using poly (acrylic acid), cellulose acetate, and polyvinyl alcohol (PVA) to prevent infection [43]. Nanomedicines are the future approach in the cure of infectious diseases. The use of metallic nanoparticles, dendrimers, polymer and lipid nanoparticles, quantum dots, and carbon nanotubes have been researched for their applications in nanomedicine. Designing and developing nanomedicines by using 3D print technology will help to satisfy the personal necessity of patients and will also offer biocompatibility (Figure 3) [45]. Thus, 3D printing is a rapid tool for manufacturing PPE to cater to the global demand, which is an alternative to the slow conventional manufacturing processes.

## 5. Current Advancements on Nanomedicine: Therapeutics and Vaccine Development

Nanotechnology is opening new therapeutic possibilities of fighting against COVID-19 by enabling new methods of prevention, diagnosis, drug-delivery, and treatment. Nanomedicine is known as the branch of medicine involved in the prevention and cure of various diseases using the nanoscale materials, such as biocompatible nanoparticles [46] and nanorobots [47], for various applications including diagnosis [48], delivery [49], sensing [50]. Nanomedicines have exhibited important features, such as efficient transport through fine capillary blood vessels and lymphatic endothelium, longer circulation duration and blood concentration, higher binding capacity to biomolecules such as endogenous compounds including proteins, higher accumulation in target tissues, reduced inflammatory or immune responses, and oxidative stress in tissues. These features vary from those of conventional medicines dependent on physiochemical properties (e.g., particle surface, size, and chemical composition) of the nanoformulations [49,51,52].

Nanomedicines specifically allow more specific drug targeting and delivery, greater safety, and biocompatibility. The more rapid development of new medicines with wide therapeutic ranges and/or improvement of in vivo pharmacokinetic properties has been reported [52]. The main purpose of nanomedicine is enhanced efficacy and reduced adverse reactions (e.g., toxicity) by altering the efficacy, safety, physicochemical properties, and pharmacokinetic/pharmacodynamic properties of the original drugs [53]. Nanomedicines have greater oral bioavailability. Longer terminal half-life can be predictable in the case of orally administered nanomedicine, which leads to a reduction of administration frequency, dose, and toxicity [53,54]. The nano delivery systems use the nanocarrier for delivering drugs at the target site. Nanocarriers (NCs) shield their load from premature degradation in the biological environment, improve bioavailability, and prolong presence in blood and cellular uptake [55]. Nanoencapsulation is the smart design of nanocarriers and are concerned with the target site and route of administration, attempting to solve the problems faced by therapeutic agents. Effective nanoparticle-based therapy includes FDA-approved lipid systems such as liposomes and micelles [56]. These liposomes and micelles can be loaded with gold or magnetic inorganic nanoparticles [57]. These properties increase the use of inorganic nanoparticles by highlighting drug delivery, imaging, and therapeutics actions. Additionally, nanoparticles help in preventing drugs from being degraded in the gastrointestinal region. They precisely support the sparing delivery of water-soluble drugs to their target location. Formulated nano drugs show higher oral bioavailability, as they display typical uptake mechanisms of absorptive endocytosis [58]. Nanoparticles such as metallic, organic, inorganic, and polymeric nanostructures, as well as dendrimers, micelles, and liposomes, are often considered in designing the target-specific drug delivery systems. Specifically, those drugs having poor solubility with less absorption ability are tagged with these nanoparticles [59]. However, polymeric nanomaterials with diameters ranging from 10 to 1000 nm show the ideal delivery vehicle [60].

### Nanotechnological Ways for Vaccine Development

Nanotechnology has caught attention as a potential strategy for the development of a new generation of vaccines, as the nanoparticles serve as a carrier for the antigen and behave as an adjuvant as well in many cases. SARS-COV and MERS treatment and vaccine candidates have not been thoroughly tested and optimized in the past due to considerably lower infection rates than COVID-19, and they have not been noted to have sufficient efficacy. In contrast to SARS or MERS, COVID-19 has been a global threat for more than a year. In research and production, innovative approaches have been recently used [61]. For SARS-CoV, MERS-CoV has been used to introduce nanotechnology into vaccines and therapeutic research on several occasions. Virus-like particles (VLPs) have recently been reported to be suitable for the development of vaccines or treatments for MERS-CoV infection symptoms [62]. Nano-sized VLPs can be delivered through the lymphatic system and capillaries in a better way than other small vaccines because they have the characteristic functions of viruses [63,64]. Additionally, they also reduce the systemic inflammatory response, and have the advantage of being able to enter cells very easily, much like the virus itself. Moreover, delivering a large number of antigens improves the antigen-presenting cell’s efficiency. As a result, the T cell receptor recognizes the synthesized complex, increasing the vaccine’s immunogenicity and efficacy [64].

VLPs that enter into the host cell are involved in B cell activation and immune system stimulation. Nano-sized VLPs have been shown to effectively overcome viruses by increasing immune response in animal experiments [65,66]. These findings were investigated for the S protein, which is found in both MERS-CoV and SARS-CoV, and hence, they can be used to effectively treat SARS-CoV-2 infection. The advantage of the present SARS-CoV-2 vaccines (approved and in the development process) is that they can be used for drug and gene delivery. The liposomes are suited to deliver nucleic acid [67].

## 6. Nanotechnology-Based Approaches in Preclinical and Clinical Studies: In Vitro and In Vivo

### 6.1. Nano-Based Approaches in Pre-Clinical Studies

COVID-19 immune-based preclinical therapeutic approaches such as virus-binding molecules; inhibitors of specific enzymes involved in viral replication and transcription; small-molecule inhibitors of helicase, proteases, or other proteins critical for the virus survival; host cell protease; endocytosis inhibitors; and siRNA inhibitors are all potential therapeutic options for SARS-CoV-2 [68]. The effects induced by monoclonal antibodies (mAb) in COVID-19 patients may also improve the development of vaccines and increasingly specific diagnostics [69]. Moreover, every single one of these tools needs to be assessed regarding clinical efficacy and safety before treating infected patients.

### 6.2. Nano-Based Approaches in Clinical Studies

Currently, nanotechnology-based formulations have been developed and commercialized for common viral infections. Several companies are moving away from conventional treatment and prevention strategies and switching over to nanotechnology for developing various types of vaccines and therapeutics, e.g., examethasones, a COVID-19 therapeutic agent that has been introduced via various nanoformulations in the treatment of COVID-19. Completing phase 3 clinical trials of Pfizer’s liposomal mRNA vaccine (BNT162b) can be considered a significant achievement in nanomedicine [70].

**Figure 4:**
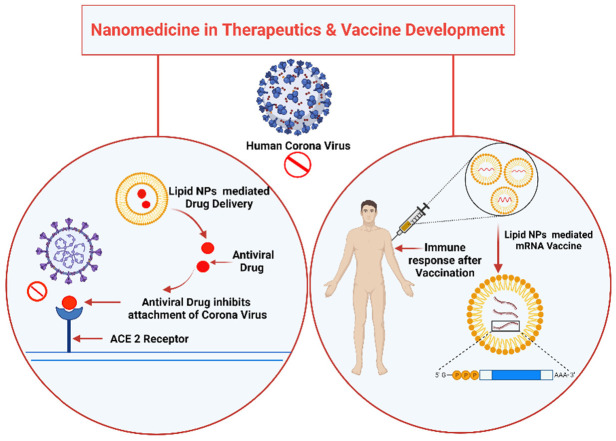
Nanomedicine in Therapeutics and Vaccine Development.

mRNA- and DNA-based vaccines would have little efficacy without nanomedicine components. According to recent research, nanomaterials may effectively inactivate SARS-CoV-2 virus, as nanomaterials have been used to inhibit viruses of other members of the Coronaviridae family [71]. Many vaccine candidates under development for the SARS-CoV-2 vaccine have safety and efficacy in the clinical and pre-clinical stages [72]. ModernaTX, Inc. used lipid nanoparticles (LNP) to encapsulate mRNA-1273, which encodes the full-length SARS-CoV-2 S protein (NCT04283461). Cells that express this viral protein will be able to present SARS-CoV-2 antigen to T cells, eliciting an immune response against the virus [73], which helps in preventing premature degradation during drug delivery. Other clinical studies are testing diverse anti-inflammatory agents to reduce lung inflammation (pneumonia), the leading cause of death in COVID-19 patients. These contain antibodies targeting inflammatory factors such as IL-6 and complement protein C5, or the CD24Fc conjugate that blocks TLR activation. There are two clinical studies that include the anti-angiogenic drug bevacizumab (anti-VEGF mAb) for reduction of lung oedema. A new antibody in clinical development is meplazumab, which blocks the binding of SARS-CoV-2 S protein to CD147 molecule on human cells, thereby reducing the virus’s infection ability. Additional immunosuppressive agents are also being tested, such as the JAK1/JAK2 inhibitor baricitinib and the antimalarial drug hydroxychloroquine sulfate. While optimal treatment regimens are still under study, different dosing and schedules are being reported by clinicians [74]. The immune response by using lipid NPs-mediated drug delivery and mRNA vaccine is shown in Figure 4.

## 7. Future Perspectives to Tackle COVID-19 Using Nanotechnology

**Figure 5:**
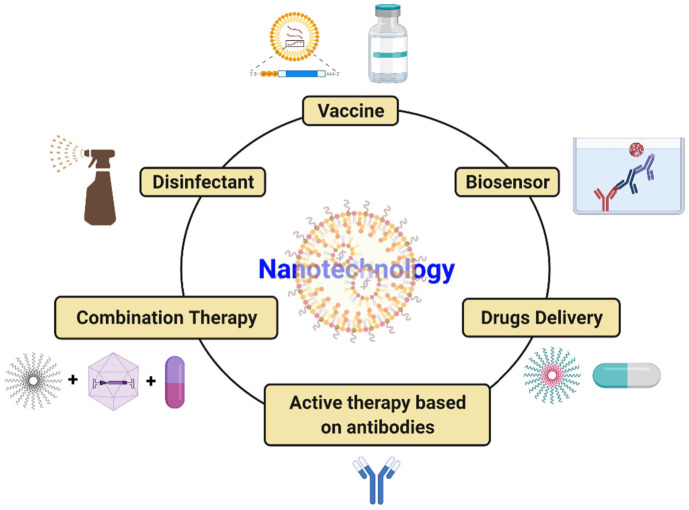
Potential strategies to tackle SARS-CoV-2 utilizing nanotechnology.

COVID-19 has introduced the scientific community to a global challenge it has perhaps never had to face before. However, it has also taught scientists and the population at large that this kind of situation could occur again. Cutting-edge tools, notably nanotechnology, should be solidly developed to tackle SARS-CoV2 infection. Nanoparticle-based medicine is a very effective tool with the potential to reduce the burden of illness. Nanoparticles that are much smaller than a micrometer have received exceptional attention in managing COVID-19 disease caused by SARS-CoV2 due to their distinctive properties (suitable size, simple preparation, minimal cost, effortless modification, etc.). Nanotechnology-based approaches for combating COVID-19 include the innovation of tools for speedy, precise, and sensitive diagnosis of SARS-CoV2 infection, production of efficient disinfectants, efficient delivery of mRNA-based vaccines into human cells, and delivery of antiviral drugs into the host. Nanotechnology is being geared up for implementation in the fight against SARS-CoV2 infection in a wide range of areas, as shown in Figure 5.

Despite the recent progress and intensive studies on nanotechnology-based tools to mitigate COVID-19, there are several important challenges remaining to be addressed when attempting to tackle COVID-19: (i) early, portable, rapid, exceedingly sensitive, and reasonable development of diagnostic kits; (ii) potential use of nanomaterials to avoid the conventional restriction associated with antiviral drugs; (iii) nanoparticle-based vaccine development to fight against SARS-CoV-2 and other pathogens; (iv) combination therapy by utilizing nanoparticles as a delivery system; (v) development of nanobiosensors for rapid and early detection of viruses; and (vi) nanomaterial-based disinfectant agents that can kill pathogens.

Some of the drawbacks associated with nanoparticles, such as cell toxicity, genotoxicity fibrosis, inflammation, immunotoxicity, and oxidative stress, are key issues to be solved before their use with patients. We anticipate that many advances will soon be accomplished in COVID-19 diagnosis, treatment, and therapy using nanotechnology-based strategies. Nanotechnology-based tools will probably be utilized in the treatment of COVID-19 and emerging pathogens. This can be achieved by nanotechnology-based therapeutic antibodies or mRNA- or protein-based vaccines, which specifically deliver the active drugs/epitopes to the host’s targeted organs and provide rapid detection of these viruses. Finally, the greatest challenge will be transferring nanomaterial technology to actual clinical applications and the feasibility of production on a large scale.

## 8. Challenges and Limitations of Nanotechnology in COVID-19

Nanotechnology-based systems, despite their benefits face numerous obstacles before they can be safely introduced to the market. Scalability and production costs are the most common issues, as are intellectual and regulatory properties and potential toxicity and environmental effects of these systems [75]. However, some bottlenecks in nanotechnology applications must be addressed before they are widely adopted in the healthcare system. The major task will be to ensure the safety of nanomaterial via in vitro studies of their biocompatibility. The fate of nanomaterials can be changed into the body when they travel through blood due to the formation of protein corona [76]. Hence, in vivo studies need to be executed carefully to better understand the toxicity of nanoparticles in the body [77]. Because of limitations, generic protocols have been employed for categorization at an early stage of research and development that minimize the chances of failures in terms of clinical translation of nanotechnology-based therapy [78]. To overcome other limitations, a closer collaboration between regulatory agencies, scientific experts in material science, pharmacology, and toxicology is required. The possible toxicity is the main concern of their use in medicine. Thus, not only the positive results of the use of nanoparticles but the appearance of unpredictable results of their action on the human body should also be investigated and scrutinized [79].

The toxicity of nanoparticles is associated with their distribution in the bloodstream and lymph streams as well as with their ability to penetrate almost all cells, tissues, and organs, as well as their ability to interact with different macromolecules. The toxicity of nanoparticles can alter the structure and functioning of organs. Nanoparticle toxicity highly depends on their physical and chemical properties, such as shape, size, surface charge, and the chemical composition of the core and shell. Several types of nanoparticles are not recognized by the body’s defense system, which may lead to the accumulation of nanoparticles in organs and tissues, leading to high toxicity or lethality. The solution is to design nanoparticles with a decreased toxicity compared with the traditional nanoparticles that are available. More advanced methods and research should be developed for studying nanoparticles’ toxicity and to analyze different pathways and mechanisms of toxicity at the molecular level [80]. Campos et al. investigated the design of nanoparticles that have small or no negative effects and concluded that it is impossible to do so unless all qualitative and quantitative physical and chemical properties of nanoparticles are systematically taken into consideration and a relevant experimental model for estimating their influence on biological systems is available [36].

## 9. Conclusions

Nanotechnology has emerged as a potential approach to the diagnosis, protection, drug delivery, and development of therapeutic strategies for controlling global pandemics such as COVID-19. Nanoparticles can serve as ideal drug carriers for pulmonary drug delivery, can be used for early and rapid detection of viruses, as part of effective treatments, and are used for nanovaccine preparation by serving as adjuvants that enhance immunogenicity and protect antigens against degradation. The functionalization of nanoparticles with versatile biomolecules and motifs that target SARS-CoV-2 would effectively develop the strategy for treatment and detection. Moreover, there are additional advantages of using nanoparticles with COVID-19 patients, particularly in hospital-acquired co-infections and superinfections caused by bacteria (*Streptococcus pneumoniae, Staphylococcus aureus, Escherichia coli, Pseudomonas aeruginosa*) and fungi (*Aspergillus* spp., *Candida* spp, *Mucor* spp., etc.). However, before using nanoparticles, their toxicity should be evaluated on experimental animals. In addition, dose dependency, the route of administration, biodistribution, and biodegradability of nanoparticles should also be considered. Finally, considering the grave situation caused by the COVID-19 pandemic, it is believed that the existing conventional platform needs to be replaced with new and emerging nanobiotechnological strategies for research on the pandemic that is caused by COVID-19 as well as in research on other related viruses.

## Data Availability

Exclude this statement.

## References

[1] Lu R., Zhao X., Li J., Niu P., Yang B., Wu H., Wang W., Song H., Huang B., Zhu N. (2020). Genomic characterisation and epidemiology of 2019 novel coronavirus: Implications for virus origins and receptor binding. Lancet.

[2] Cevik M., Kuppalli K., Kindrachuk J., Peiris M. (2020). Virology, transmission, and pathogenesis of SARS-CoV-2. BMJ.

[3] Tang Z., Zhang X., Shu Y., Guo M., Zhang H., Tao W. (2020). Insights from nanotechnology in COVID-19 treatment. Nano Today.

[4] Rangayasami A., Kannan K., Murugesan S., Radhika D., Sadasivuni K.K., Reddy K.R., Raghu A.V. (2021). Influence of nanotechnology to combat against COVID-19 for global health emergency: A review. Sens. Int..

[5] Honardoost M., Jananic L., Aghilia R., Emamia Z., Khamseh M.E. (2021). The Association between Presence of Comorbidities and COVID-19 Severity: A Systematic Review and Meta-Analysis. Cerebrovasc. Dis..

[6] Biswas M., Rahaman S., Biswas T.K., Haque Z., Ibrahim B. (2021). Association of sex, age, and comorbidities with mortality in COVID-19 patients: A systematic review and meta-analysis. Intervirology.

[7] Nishiga M., Wang D.W., Han Y., Lewis D.B., Wu J.C. (2020). COVID-19 and cardiovascular disease: From basic mechanisms to clinical perspectives. Nat. Rev. Cardiol..

[8] Abu-Farha M., Al-Mulla F., Thanaraj T.A., Kavalakatt S., Ali H., Ghani M.A., Abubaker J. (2020). Impact of diabetes in patients diagnosed with COVID-19. Front. Immunol..

[9] Grivas P., Khaki A.R., Wise-Draper T.M., French B., Hennessy C., Hsu C.-Y., Shyr Y., Li X., Choueiri T.K., Painter C.A. (2021). Association of clinical factors and recent anticancer therapy with COVID-19 severity among patients with cancer: A report from the COVID-19 and Cancer Consortium. Ann. Oncol.

[10] Wang C., Zhang M., Garcia G., Tian E., Cui Q., Chen X., Sun G., Wang J., Arumugaswami V., Shi Y. (2021). ApoE-isoform-dependent SARS-CoV-2 neurotropism and cellular response. Cell. Stem. Cell..

[11] Wang Q.Q., Davis P.B., Gurney M.E., Xu R. (2021). COVID-19 and dementia: Analyses of risk, disparity, and outcomes from electronic health records in the US. Alzheimer’s Dement.

[12] Yu I.T.S., Li Y., Wong T.W., Tam W., Chan A.T., Lee J.H.W., Leung D.Y.C., Tommy Ho B.S. (2004). Evidence of Airborne Transmission of the Severe Acute Respiratory Syndrome Virus. N. Engl. J. Med..

[13] Otter J.A., Donskey C., Yezli S., Douthwaite S., Goldenberg S.D., Weber D.J. (2016). Transmission of SARS and MERS coronaviruses and influenza virus in healthcare settings: The possible role of dry surface contamination. J. Hosp. Infect.

[14] Li Q., Hu C., Haerter R., Englert U. (2004). Isosteric molecules in non-isomorphous structures: A new route to new structures. The example of 9,10-dihaloanthracene. CrystEngComm.

[15] Kuba K., Imai Y., Rao S., Gao H., Guo F., Guan B., Huan Y., Yang P., Zhang Y., Deng W. (2005). A crucial role of angiotensin converting enzyme 2 (ACE2) in SARS coronavirus–induced lung injury. Nat. Med..

[16] Jia H.P., Look D.C., Shi L., Hickey M., Pewe L., Netland J., Farzan M., Wohlford-Lenane C., Perlman S., McCray P.B. (2005). ACE2 receptor expression and severe acute respiratory syndrome coronavirus infection depend on differentiation of human airway epithelia. J. Virol..

[17] Hamming I., Timens W., Bulthuis M.L.C., Lely A.T., Navis G.J., Van Goor H. (2004). Tissue distribution of ACE2 protein, the functional receptor for SARS coronavirus. A first step in understanding SARS pathogenesis. J. Pathol..

[18] Ziegler C.G.K., Allon S.J., Nyquist S.K., Mbano I.M., Miao V.N., Tzouanas C.N., Tzouanas C.N., Cao Y., Yousif A.S., Bals J. (2020). SARS-CoV-2 receptor ACE2 is an interferon-stimulated gene in human airway epithelial cells and is detected in specific cell subsets across tissues. Cell.

[19] Wang D., Hu B., Hu C., Zhu F., Liu X., Zhang J., Wang B., Xiang H., Cheng Z., Xiong Y. (2020). Clinical Characteristics of 138 Hospitalized Patients With 2019 Novel Coronavirus–Infected Pneumonia in Wuhan, China. JAMA.

[20] Chan J.F.W., Yuan S., Kok K.H., To K.K.W., Chu H., Yang J., Xing F., Nurs J.L.B., Yip C.C.-Y., Poon R.W.-S. (2020). A familial cluster of pneumonia associated with the 2019 novel coronavirus indicating person-to-person transmission: A study of a family cluster. Lancet.

[21] Ksiazek T.G., Erdman D., Goldsmith C.S., Zaki S.R., Peret T., Emery S., Tong S., Urbani C., Comer J.A., Lim M.P.H.W. (2003). A novel coronavirus associated with severe acute respiratory syndrome. N. Engl. J. Med..

[22] Guery B., Poissy J., elMansouf L., Séjourné C., Ettahar N., Lemaire X., Vuotto F., Goffard A., PharmD S.B., Enouf V. (2013). Clinical features and viral diagnosis of two cases of infection with Middle East Respiratory Syndrome coronavirus: A report of nosocomial transmission. Lancet.

[23] Totura A.L., Baric R.S. (2012). SARS coronavirus pathogenesis: Host innate immune responses and viral antagonism of interferon. Curr. Opin. Virol..

[24] Hadjadj J., Yatim N., Barnabei L., Corneau A., Boussier J., Smith N., Péré H., Charbit B., Bondet V., Chenevier-Gobeaux C. (2020). Impaired type I interferon activity and exacerbated inflammatory responses in severe Covid-19 patients. MedRxiv.

[25] Blanco-Melo D., Nilsson-Payant B.E., Liu W.C., Uhl S., Hoagland D., Møller R., Jordan T.X., Oishi K., Panis M., Sachs D. (2020). Imbalanced host response to SARS-CoV-2 drives development of COVID-19. Cell.

[26] Huang C., Wang Y., Li X., Ren L., Zhao J., Hu Y., Zhang P.L., Fan G., Xu J., Gu X. (2020). Clinical features of patients infected with 2019 novel coronavirus in Wuhan, China. Lancet.

[27] Jansen J.M., Gerlach T., Elbahesh H., Rimmelzwaan G.F., Saletti G. (2019). Influenza virus-specific CD4+ and CD8+ T cell-mediated immunity induced by infection and vaccination. J. Clin. Virol..

[28] Behrens E.M., Koretzky G.A. (2017). Cytokine storm syndrome: Looking toward the precision medicine era. Arthritis Rheumatol..

[29] Alhalaili B., Popescu I.N., Kamoun O., Alzubi F., Alawadhia S., Vidu R. (2020). Nanobiosensors for the detection of novel coronavirus 2019-nCoV and other pandemic/Epidemic Respiratory viruses: A review. Sensors.

[30] Antiochia R. (2020). Nanobiosensors as new diagnostic tools for SARS, MERS and COVID-19: From past to perspectives. Microchim. Acta.

[31] Jindal S., Gopinath P. (2020). Nanotechnology based approaches for combating COVID-19 viral infection. Nano Express.

[32] Martinez-Paredes G., Gonzalez-Garcia M.B., Costa-Garcia A. (2009). Genosensor for SARS virus detection based on gold nanostructured screen-printed carbon electrodes. Electroanal.

[33] Iravani S. (2020). Nano-and biosensors for the detection of SARS-CoV-2: Challenges and opportunities. Mater. Adv..

[34] Ahmed S.R., Nagy E., Neethirajan S. (2017). Self-assembled star-shaped chiroplasmonic gold nanoparticles for an ultrasensitive chiroimmunosensor for viruses. RSC Adv..

[35] Shiaelis N., Tometzki A., Peto L., McMahon A., Hepp C., Bickerton E., Favard C., Muriaux D., Andersson M., Oakley S. (2021). Virus detection and identification in minutes using single-particle imaging and deep learning. MedRxiv.

[36] Campos E.V.R., Pereira A.E.S., de Oliveira J.L., Carvalho L.B., Guilger-Casagrande M., de Lima R., Fraceto L.F. (2020). How can nanotechnology help to combat COVID-19? Opportunities and urgent need. J. Nanobio..

[37] Bhattacharjee S., Joshi R.K., Chughtai A.A., Macintyre C.R. (2019). Graphene modified multifunctional personal protective clothing. Adv. Mater. Interfaces.

[38] Chaudhary V., Royal A., Chavali M., Yadav S.K. (2021). Advancements in Research and development to combat COVID-19 using Nanotechnology. Nanotech. Environ. Eng..

[39] Talebian S., Wallace G.G., Schroeder A., Stellacci F., Conde J. (2020). Nanotechnology-based disinfectants and sensors for SARS-CoV-2. Nat. Nanotechnol..

[40] Shirvanimoghaddam K., Akbari M.K., Yadav R., Al-Tamimi A.K., Naebe M. (2021). Fight against COVID-19: The case of antiviral surfaces. APL Mater..

[41] Basak S., Packirisamy G. (2020). Nano-based antiviral coatings to combat viral infections. Nanostruct. Nanoobjects.

[42] Erkoc P., Ulucan-Karnak F. (2021). Nanotechnology-based antimicrobial and antiviral surface coating strategies. Prosthesis.

[43] Aydin A., Demirtas Z., Ok M., Erkus H., Cebi G., Uysal E., Gunduz O., Ustundag C.B. (2021). 3D printing in the battle against COVID-19. Emerg. Mater..

[44] Oladapo B.I., Ismail S.O., Afolalu T.D., Olawade D.B., Zahedi M. (2021). Review on 3D printing: Fight against COVID-19. Mat. Chem. Phys..

[45] Jain K., Shukla R., Yadav A., Ujjwal R.R., Flora S.J.S. (2021). 3D printing in development of Nanomedicine. Nanomater.

[46] McNamara K., Tofail S.A. (2015). Nanosystems: The use of nanoalloys, metallic, bimetallic, and magnetic nanoparticles in biomedical applications. Phys. Chem. Chem. Phys..

[47] Saadeh Y., Vyas D. (2014). Nanorobotic applications in medicine: Current proposals and designs. Am. J. Robot. Surg..

[48] Oliveira O.N., Iost R.M., Siqueira J.R., Crespilho F.N., Caseli L. (2014). Nanomaterials for diagnosis: Challenges and applications in smart devices based on molecular recognition. ACS Appl. Mater. Interfaces.

[49] De Jong W.H., Borm P.J. (2008). Drug delivery and nanoparticles: Applications and hazards. Int. J. Nanomed..

[50] Holzinger M., Le Goff A., Cosnier S. (2014). Nanomaterials for biosensing applications: A review. Front. Chem..

[51] Liu W., Yang X.L., Ho W.S. (2011). Preparation of uniform-sized multiple emulsions and micro/nano particulates for drug delivery by membrane emulsification. J. Pharm. Sci..

[52] Onoue S., Yamada S., Chan H.K. (2014). Nanodrugs: Pharmacokinetics and safety. Int. J. Nanomed..

[53] Dawidczyk C.M., Kim C., Park J.H., Russell L.M., Lee K.H., Pomper M.G., Searson P.C. (2014). State-of-the-art in design rules for drug delivery platforms: Lessons learned from FDA-approved nanomedicines. J. Control. Release.

[54] Charlene M.D., Luisa M.R., Peter C.S. (2014). Nanomedicines for cancer therapy: State-of-the-art and limitations to pre-clinical studies that hinder future developments. Front. Chem..

[55] Kumari A., Yadav S.K. (2011). Cellular interactions of therapeutically delivered nanoparticles. Exp. Opinion Drug. Deliv..

[56] Shi X., Sun K., Baker J.R. (2008). Spontaneous formation of functionalized dendrimer-stabilized gold nanoparticles. J. Phys. Chem. C..

[57] Park S.H., Oh S.G., Mun J.Y., Han S.S. (2006). Loading of gold nanoparticles inside the DPPC bilayers of liposome and their effects on membrane fluidities. Coll. Surf. B.

[58] Patra J.K., Das G., Fraceto L.F., Campos E.V., del Pilar Rodriguez-Torres M., Acosta-Torres L.S., Diaz-Torres L.A., Grillo R., Swamy M.K., Sharma S. (2018). Nano based drug delivery systems: Recent developments and future prospects. J. Nanobiotechnol..

[59] Behera S., Rana G., Satapathy S., Mohanty M., Pradhan S., Panda M.K., Ningthoujam R., Hazarika B.N., Singh Y.D. (2020). Biosensors in diagnosing COVID-19 and recent development. Sens. Internat..

[60] Bonifácio B.V., da Silva P.B., dos Santos Ramos M.A., Negri K.M.S., Bauab T.M., Chorilli M. (2014). Nanotechnology-based drug delivery systems and herbal medicines: A review. Int. J. Nanomed..

[61] Chakravarty M., Vora A. (2014). Drug Delivery and Translational Research. Drug. Deliv. Transl. Res..

[62] Reddy S.T., Van der Vlies A.J., Simeoni E., Angeli V., Randolph G.J., O’Neil C.P., Lee L.K., Swartz M.A., Hubbell J.A. (2007). Exploiting lymphatic transport and complement activation in nanoparticle vaccines. Nat. Biotechnol..

[63] Reddy S.T., Berk D.A., Jain R.K., Swartz M.A. (2006). A sensitive in vivo model for quantifying interstitial convective transport of injected macromolecules and nanoparticles. J. Appl. Physiol..

[64] Bachmann M.F., Jennings G.T. (2010). Vaccine delivery: A matter of size, geometry, kinetics and molecular patterns. Nat. Rev. Immunol..

[65] Ma C.Q., Wang L.L., Tao X.R., Zhang N., Yang Y., Tseng C.-T.K., Li F., Zhou Y., Jiang S., Dua L. (2014). Searching for an ideal vaccine candidate among different MERS coronavirus receptor-binding fragments-The importance of immune focusing in subunit vaccine design. Vaccine.

[66] Wang L.S., Shi W., Joyce M.G., Modjarrad K., Zhang Y., Leung K., Lees C.R., Zhou T., Yassine H.M., Kanekiyo M. (2015). Evaluation of candidate vaccine approaches for MERS-CoV. Nat. Commun..

[67] Milane L., Amiji M. (2021). Clinical approval of nanotechnology-based SARS-CoV-2 mRNA vaccines: Impact on translational nanomedicine. Drug. Deliv. Transl. Res..

[68] Dhama K., Sharun K., Tiwari R., Dadar M., Malik Y.S., Singh K.P., Chaicumpa W. (2020). COVID-19, an emerging coronavirus infection: Advances and prospects in designing and developing vaccines, immunotherapeutics, and therapeutics. Hum. Vaccines Immunother..

[69] Marston H.D., Paules C.I., Fauci A.S. (2018). Monoclonal Antibodies for Emerging Infectious Diseases-Borrowing from History. N. Engl. J. Med..

[70] Rab S., Afjal A., Javaid M., Haleem A., Vaishya R. (2020). An update on the global vaccine development for coronavirus. Diabetes Metab. Syndr..

[71] Chen Y.N., Hsueh Y.H., Hsieh C.T., Tzou D.Y., Chang P.L. (2016). Antiviral Activity of Graphene–Silver Nanocomposites against Non-Enveloped and Enveloped Viruses. Int. J. Environ. Res. Public Health.

[72] Florindo H.F., Kleiner R., Vaskovich-Koubi D., Acúrcio R.C., Carreira B., Yeini E., Tiram G., Liubomirski Y., Satchi-Fainaro R. (2020). Immune-mediated approaches against COVID-19. Nat. Nanotechnol..

[73] Shah V.K., Firmal P., Alam A., Ganguly D., Chattopadhyay S. (2020). Overview of Immune Response During SARS-CoV-2 Infection: Lessons From the Past. Front. Immunol..

[74] Fleischmann R., Genovese M.C., Lin Y., St John G., Van der Heijde D., Wang S., Gomez-Reino J.J., Maldonado-Cocco J.A., Stanislav M., Kivitz A.J. (2020). Long-term safety of sarilumab in rheumatoid arthritis: An integrated analysis with up to 7 years’ follow-up. Rheumatology.

[75] Chan W.C.W. (2020). Nano research for COVID-19. ACS Nano..

[76] Polyak B., Cordovez B. (2016). How can we predict behavior of nanoparticles in vivo?. Nanomedicine.

[77] Beyth N., Houri-Haddad Y., Domb A., Khan W., Hazan R. (2015). Alternative antimicrobial approach: Nano-antimicrobial materials. Evid. Based Complement. Altern. Med..

[78] Ventola C.L. (2017). Progress in nanomedicine: Approved and investigational nanodrugs. Pharm. Ther..

[79] Caster J.M., Patel A.N., Zhang T., Wang A. (2017). Investigational nanomedicines in 2016: A review of nanotherapeutics currently undergoing clinical trials. Wiley Interdiscip. Rev. Nanomed. Nanobiotechnol..

[80] Sukhanova A., Bozrova S., Sokolov P., Berestovoy M., Karaulov A., Nabiev I. (2018). Dependence of Nanoparticle Toxicity on Their Physical and Chemical Properties. Nanoscale Res. Lett..

